# An Efficient, Mild and Solvent-Free Synthesis of Benzene Ring Acylated Harmalines

**DOI:** 10.3390/molecules15010068

**Published:** 2009-12-28

**Authors:** Sabira Begum, Farhat Zubair, Syed Nawazish Ali, Bina Shaheen Siddiqui

**Affiliations:** H.E.J. Research Institute of Chemistry, International Center for Chemical and Biological Sciences, University of Karachi, Karachi-75270, Pakistan; E-Mails: farhatzubair@yahoo.com (F.); nawazishalii@yahoo.com (S.N.A.); bina@comsats.net.pk (B.S.S.)

**Keywords:** β-carboline, acylharmaline, Friedel-Crafts acylation, solvent-free reaction

## Abstract

A facile synthesis of a series of benzene ring acylated analogues of harmaline has been achieved by Friedel-Crafts acylation under solvent-free conditions at room temperature using acyl halides/acid anhydrides and AlCl_3_. The reaction afforded 10- and 12-acyl analogues of harmaline in good yield, along with minor quantities of *N*-acyl-tryptamines and 8-acyl analogues of *N*-acyltryptamines.

## Introduction

The Harmala alkaloids are β-carboline system derivatives which frequently occur in the indole alkaloid series. Harmine and harmaline are the main alkaloids of *Peganum harmala* (Zygophylaceae) seeds [1,2,3]. These alkaloids are valued for their interesting chemistry, pharmacological importance and therapeutic potential. They possess antitumour, antileishmanial, anti-HIV and other important activities [4,5,6,7]. The existence of imine-enamine equilibrium in harmaline was established through ^1^H-NMR studies to be strongly displaced towards the imino form [8,9,10,11]. Various *C*- and *N*-alkylated and acylated products of harmaline may be satisfactorily interpreted through this tautomerism [9,12]. 

Chemical modifications of natural product have been the major means to explore more potent analogues. The Friedel-Crafts acylation is an important method for the preparation of aromatic ketones by the reaction of aromatic substrate with acylating agent in the presence of Lewis acid catalyst. The optimization of these preparative processes is of great importance due to the considerable practical value of the aromatic ketone products as these compounds constitute fundamental intermediates in the pharmaceutical, fragrance, flavour, dye and agrochemical industries [13,14,15]. 

Due to increasing environmental concerns, the solvent-free chemical synthesis has received much attention now a day. These processes are much appealing as they are environmentally benign, economical and provide an opportunity to work with an open vessel requiring short reaction time and simple work up [16,17,18,19]. We have previously reported the Friedel-Crafts acylation of *N*-acetyl tetrahydroharmine under solvent-free conditions which resulted in synthesis of a series of its 10-acyl and 12-acyl analogues in high overall yields [20]. As a continuation of our studies in this direction, we have attempted the reaction of harmaline (**1**) with Friedel-Crafts reagents (acyl halides/acid anhydrides and AlCl_3_) at room temperature and under solvent-free conditions. As a result 10-acyl (**2**−**10**) and 12-acyl (**11**−**19**) analogues of harmaline were obtained in high overall yield along with several *N*-acyl tryptamines (**20**−**28**) and 8-acyl analogues of *N*-acyltryptamines (**29**−**31**) as minor products. All compounds were characterized with the help of spectral studies. Except **for **compound **20**, all derivatives are new. 

## Results and Discussion

In order to investigate whether harmaline undergoes acylation conveniently on the benzene ring in the presence of the sensitive imine-enamine functionality in the present studies Friedel-Crafts acylation under solvent-free conditions was undertaken. Friedel-Crafts reaction on **1 **was carried out with the following reagents: acetic anhydride, propanoyl chloride, butyric anhydride, valeryl chloride, hexanoyl chloride, heptanoyl chloride, octanoyl chloride, nonanoyl chloride and decanoyl chloride (entries **A** to **I**) in the presence of AlCl_3_ under solvent-free conditions (*vide* Experimental). As a result 10-acyl (**2**−**10**) and 12-acyl (**11**−**19**) derivatives of **1** were obtained in high overall yield, along with minor quantities of the *N*-acyl (**20**−**28**) and 8,*N-*diacyl tryptamines (**29**−**31**) resulting from pyrido ring opening (Scheme 1). It may be noted that by changing the quantity of the catalyst either the *N*-acyl or *aromatic*-acyl product was formed as the major product, as discussed below.

The *N*-acyl tryptamine derivatives **20**−**28** were obtained in high yield when a lower quantity of AlCl_3_ (~200 mg) was used. Under these conditions the reaction further afforded minor quantities of the 8-acyl analogues of *N*-acyltryptamines **29**−**31**. The pyrido ring opening during acylation was reported earlier [10]. However, on increasing the quantity of catalyst (~400 mg) the results were in favour of aromatic acylation leading to 10-acyl (**2**−**10**) and 12-acyl (**11**−**19**) analogues of harmaline with minor quantities of **20**–**31**. Grinding the catalyst first with harmaline for a few minutes also reduces *N*-acylation due to catalyst’s association with imino nitrogen. It is also worth noting that the 10-acylated regioisomers **2**−**10** were obtained in good yield on increasing the carbon chain in the acylating reagents (Table 1, entries **C** to **I**).

**Table 1:** Friedel-Crafts Acylation of Harmaline (**1**) under Solvent-Free Conditions.

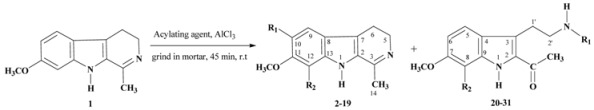							
Entry	Acylating reagents	10-Acylharmaline	Yield **	12-Acylharmaline	Yield **	N-Acyltryptamine product	Yield **
		(R_1_=), (R_2 _=H)	(%) *^a^*	(R_1_=H), (R_2 _=)	(%) *^a^*	(R_1_=), (R_2_ =H)	(%) *^a^*
**A**	(CH_3_-CO)_2_O	**2**) ^1'^CO-^2'^CH_3_	21.4	**11**) ^1'^CO-^2'^CH_3_	41.6	**20**) ^1''^CO-^2''^CH_3_	4.3
**B**	CH_3_-CH_2_-CO-Cl	**3**) ^1^^'^CO-^2^^'^CH_2_-^3^^'^CH_3_	24.6	**12**) ^1^^'^CO-^2^^'^CH_2_-^3^^'^CH_3_	40.6	**21**) ^1''^CO-^2''^CH_2_-^3''^CH_3_	5.1
**C**	(CH_3_-(CH_2_)_2_-CO)_2_O	**4**) ^1^^'^CO-(CH_2_)_2_-^4^^'^CH_3_	38.7	**13**) ^1^^'^CO-(CH_2_)_2_-^4^^'^CH_3_	20.4	**22**) ^1''^CO-(CH_2_)_2_-^4''^CH_3_	4.6
**D**	CH_3_-(CH_2_)_3_-CO-Cl	**5**) ^1^^'^CO-(CH_2_)_3_-^5^^'^CH_3_	40.0	**14**) ^1^^'^CO-(CH_2_)_3_-^5^^'^CH_3_	18.5	**23**) ^1''^CO-(CH_2_)_3_-^5''^CH_3_	5.0
**E**	CH_3_-(CH_2_)_4_-CO-Cl	**6**) ^1^^'^CO-(CH_2_)_4-_^6^^'^CH_3_	40.8	**15**) ^1^^'^CO-(CH_2_)_4-_^6^^'^CH_3_	17.4	**24**) ^1''^CO-(CH_2_)_4_-^6''^CH_3_	3.8
**F**	CH_3_-(CH_2_)_5_-CO-Cl	**7**) ^1^^'^CO-(CH_2_)_5_-^7^^'^CH_3_	41.6	**16**) ^1^^'^CO-(CH_2_)_5_-^7^^'^CH_3_	16.8	**25**) ^1''^CO-(CH_2_)_5_-^7''^CH_3_	5.4
**G**	CH_3_-(CH_2_)_6_-CO-Cl	**8**) ^1^^'^CO-(CH_2_)_6_-^8^^'^CH_3_	42.4	**17**) ^1^^'^CO-(CH_2_)_6_-^8^^'^CH	17.0	**26**) ^1''^CO-(CH_2_)_6-_^8''^CH_3_	4.5
**H**	CH_3_-(CH_2_)_7_-CO-Cl	**9**) ^1^^'^CO-(CH_2_)_7_-^9^^'^CH_3_	43.5	**18**) ^1^^'^CO-(CH_2_)_7_-^9^^'^CH_3_	16.6	**27**) ^1''^CO-(CH_2_)_7-_^9''^CH_3_	5.6
**I**	CH_3_-(CH_2_)_8_-CO-Cl	**10**) ^1^^'^CO-(CH_2_)_8_-^10^^'^CH_3_	44.0	**19**) ^1^^'^CO-(CH_2_)_8_-^10^^'^CH_3_	15.8	**28**) ^1''^CO-(CH_2_)_8-_^10''^CH_3_	5.2

*Notes*: 8,*N*-Diacyltryptamine products (**R_1_** = **R_2_** = acyl; **29**, **30** and **31**) were also obtained as minor products (yield: 3.2, 3.5 and 4.0% respectively) in the entries **G**, **H** and **I**; ^a ^Isolated yield of products.

**Figure 1:**
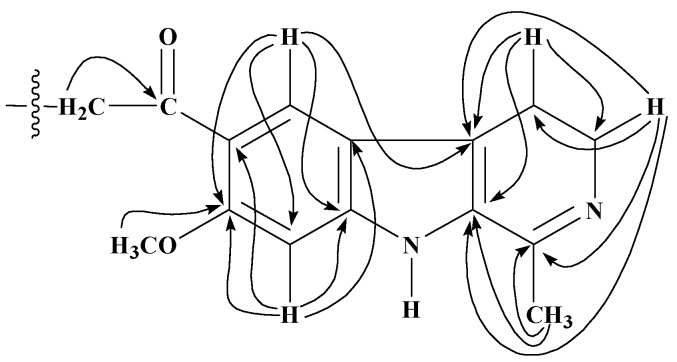
Significant HMBC (^1^H 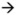 ^13^C) interactions of 10-acyl analogues of **1**.

**Figure 2:**
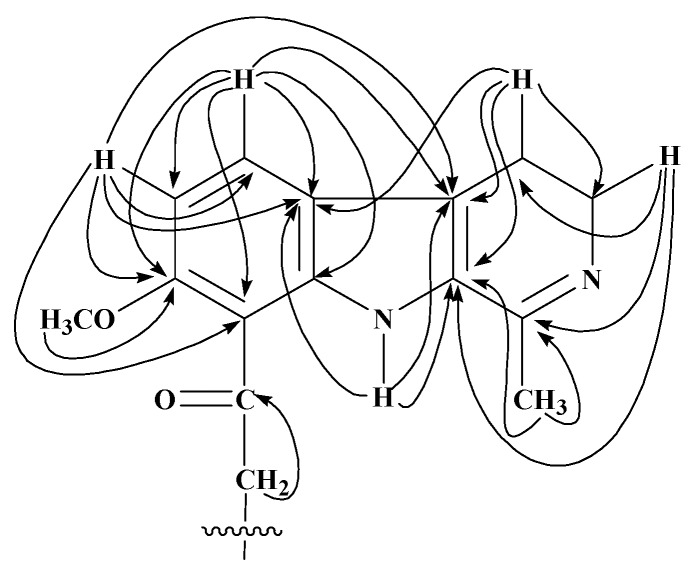
Significant HMBC (^1^H 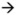 ^13^C) interactions of 12-acyl analogues of **1**.

**Figure 3:**
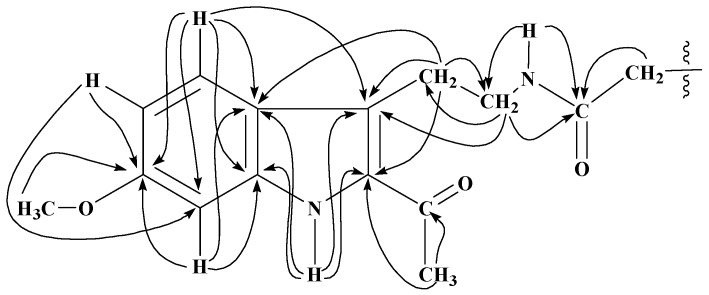
Significant HMBC (^1^H 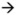 ^13^C) interactions of *N*-acylated tryptamine analogues obtained from **1**.

**Figure 4:**
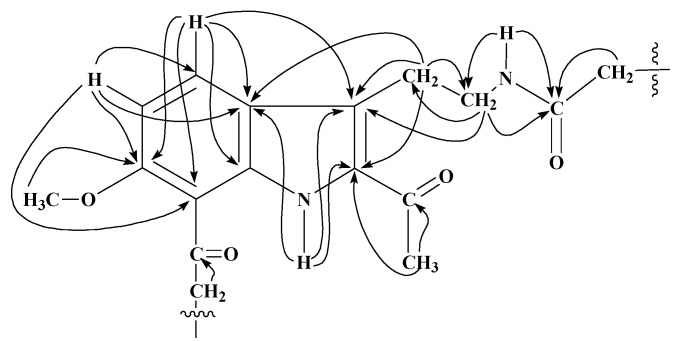
Significant HMBC (^1^H 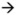 ^13^C) interactions of 8, *N*-diacyl analogues of tryptamine obtained from **1**.

These derivatives have been characterized by spectral studies including IR, EIMS, HREIMS, 1D, (^1^H-NMR and ^13^C-NMR; Broad Band decoupled, DEPT) and 2D-NMR (^1^H,^1^H COSY, TOCSY, HMQC and HMBC) (see Experimental) and comparison of spectral data with reported values of similar compounds [20,21,22,23,24,25]. For the 10-acyl analogues of harmaline **2**−**10**, the presence of two singlets at ~δ 7.9 and 6.9 in the ^1^H-NMR spectra assignable to H-9 and H-12 respectively was in accord with substitution on C-10 position of harmaline. The ^1^H-NMR spectra of 12-acyl analogues **11**−**19** displayed two characteristic sets of one proton doublets at ~δ 7.7 (*J* = 8.8 Hz) and 6.9 (*J* = 8.8 Hz) assigned to H-9 and H-10 respectively, showing the substitution on C-12. For compounds **20**−**28**, characteristic peaks of H-5, H-6 and H-8 appeared in the ^1^H-NMR spectra as one proton doublets, double doublets and doublets at ~δ 7.55 (*J* = 8.8 Hz), 6.79 (*J* = 8.8 and 2.0 Hz) and 6.75 (*J* = 2.0 Hz), respectively, suggesting no acylation on benzene ring. However, the presence of a triplet and a quartet at δ 3.28 (*J* = 6.9 Hz) and 3.54 (*J* = 6.9) assignable to H-1′ and H-2′ respectively of *N*-acylamidoethyl group and a three-proton sharp singlet for 2-acetyl group at δ 2.61 were highly suggestive of *N*-acylation and subsequent pyrido ring opening of harmaline during aqueous workup. Molecular ion peaks in the EIMS and HREIMS also confirmed these results. The ^1^H-NMR spectra of compounds **29**−**31** showed substitution both on benzene ring and pyrido nitrogen and concomitant pyrido ring opening. A set of two mutually coupled downfield doublets at δ 7.86 and 6.88 with a 8.8 Hz coupling constant clearly indicated substitution on C-8 of tryptamine which corresponds to C-12 in harmaline. Further the mass and ^1^H-NMR analysis of crude reaction products indicated the presence of corresponding 6,*N*-diacyltryptamine derivatives also, which could not be separated in the present studies. Formation of 6,*N*-diacyl and 8,*N*-diacyltryptamine derivatives in minor quantities indicates that the 2-acetyl group in tryptamine deactivated the benzene ring. The ^13^C-NMR signals of quaternary carbons were particularly assigned on the basis of HMBC connectivities observed for these carbons with various protons which are shown in Figure 1 and Figure 2 for 10- and 12-acyl analogues of harmaline, and Figure 3 and Figure 4 for *N*-acyl and 8, *N*-diacyl analogues of tryptamine respectively.

## Experimental

### General

The melting points were determined using a Buchi-535 melting point apparatus and are uncorrected. Infrared spectra were recorded on a Bruker VECTOR 22 spectrophotometer. The ^1^H- and ^13^C-NMR spectra were recorded on a Bruker Avance 400 spectrometer operating at 400 MHz (^1^H) and 100 MHz (^13^C). Mass spectra were run on a Jeol JMS-HX110 (high-resolution, E.I. probe, 70 eV) and a Varian MAT 311 A (low resolution, E.I. probe, 70 eV) instrument. Harmaline (**1**) used in the present studies was isolated from the seeds of *P. harmala* using the procedure described by Siddiqui [26].

### General Procedure for Solvent-Free Friedel-Crafts Acylation

A mixture of **1** (100 mg, 0.47 mmol), acylating agent (2.5 mL) and anhydrous AlCl_3_ (400 mg, 3.00 mmol) was thoroughly ground in an agate mortar and pestle for 45 min in fume cupboard and then kept at room temperature for 1 h. The reaction mixture was then poured into crushed ice, basified with 30% NH_3_ and extracted with EtOAc. The EtOAc layer was washed with water, dried (Na_2_SO_4_) and freed of solvent under reduced pressure. A solid mass thus obtained afforded compounds **2**−**31** (Table 1) through chromatographic procedures including column chromatography (silica gel; Merck 9385; CHCl_3_-MeOH in increasing order of polarity from 9.95:0.05 to 8.25:1.25) and subsequent preparative TLC (Kieselgel 60 F_254_ precoated aluminium cards; Merck, 9.9:0.1 to 9.5:0.5 CHCl_3_-MeOH).

*10-Acetyl-11-methoxy-3-methyl-5, 6-dihydro-β-carboline* (**2**). Off white crystals (MeOH); mp 261–262 °C; IR (KBr) ν_max_: 3,418 (indole N-H), 1,668 (ketone C=O) cm^-1^; ^1^H-NMR (CDCl_3_): δ 7.89 (1H, s, H-9), 6.84 (1H, s, H-12), 3.88 (3H, s, OCH_3_), 3.80 (2H, t, *J* = 8.4 Hz, H-5), 2.84 (2H, t, *J* = 8.4 Hz, H-6), 2.70 (3H, s, H-2'), 2.36 (3H, s, H-14); ^13^C-NMR (CDCl_3_): δ 201.2 (C-1'), 157.9* (C-11), 157.7* (C-3), 140.1 (C-13), 129.4 (C-2), 124.8 (C-8), 123.5 (C-9), 119.9 (C-10), 119.1 (C-7), 93.7 (C-12), 55.8 (OCH_3_), 47.6 (C-5), 32.5 (C-2´), 21.6 (C-14), 19.3 (C-6); EIMS *m/z* (rel. int.%): 256 [M^+^] (30), 241 (100), 239 (47), 226 (6), 198 (6), 182 (22); HREIMS Calcd. for [C_15_H_16_N_2_O_2_]: 256.1212. Found: 256.1218.

*10-Propionyl-11-methoxy-3-methyl-5, 6-dihydro-β-carboline* (**3**). Off white crystals (MeOH); mp 263–264 °C; IR (KBr) ν_max_: 3,419 (indole N-H), 1,664 (ketone C=O) cm^-1^; ^1^H-NMR (CDCl_3_): δ 7.95 (1H, s, H-9), 6.85 (1H, s, H-12), 3.90 (3H, s, OCH_3_), 3.80 (2H, t, *J* = 8.4 Hz, H-5), 3.00 (2H, q, *J* = 7.2 Hz, H-2'), 2.82 (2H, t, *J* = 8.4 Hz, H-6), 2.35 (3H, s, H-14), 1.16 (3H, t, *J* = 7.2 Hz, H-3'); ^13^C-NMR (CDCl_3_): δ 203.4 (C-1'), 157.6 (C-3 and C-11), 140.0 (C-13), 129.2 (C-2), 124.8 (C-8), 123.3 (C-9), 119.7 (C-10), 119.0 (C-7), 93.6 (C-12), 55.7 (OCH_3_), 47.5 (C-5), 36.8 (C-2'), 21.5 (C-14), 19.3 (C-6), 8.8 (C-3'); EIMS *m/z* (rel. int.%): 270 [M^+^] (63), 241 (100), 226 (8), 198 (5), 182 (12); HREIMS Calcd. for [C_16_H_18_N_2_O_2_]: 270.1368. Found: 270.1370.

*10-Butyryl-11-methoxy-3-methyl-5,6-dihydro-β-carboline* (**4**). Off white crystals (MeOH); mp 266–267 °C; IR (KBr) ν_max_: 3,417 (indole N-H), 1,662 (ketone C=O) cm^-1^; ^1^H-NMR (CDCl_3_): δ 7.92 (1H, s, H-9), 6.84 (1H, s, H-12), 3.88 (3H, s, OCH_3_), 3.80 (2H, t, *J* = 8.4 Hz, H-5), 2.96 (2H, t, *J* = 7.4 Hz, H-2'), 2.81 (2H, t, *J* = 8.4 Hz, H-6), 2.33 (3H, s, H-14), 1.70 (2H, sextet, *J* = 7.4 Hz, H-3'), 0.95 (3H, t, *J* = 7.4 Hz, H-4'); ^13^C-NMR (CDCl_3_): δ 203.2 (C-1'), 157.7* (C-3), 157.5* (C-11), 140.0 (C-13), 129.2 (C-2), 124.9 (C-8), 123.2 (C-9), 119.6 (C-10), 118.9 (C-7), 93.6 (C-12), 55.7 (OCH_3_), 47.5 (C-5), 45.6 (C-2'), 21.6 (C-14), 19.3 (C-6), 18.2 (C-3'), 14.0 (C-4'); EIMS *m/z* (rel. int.%): 284 [M^+^] (79), 256 (2), 241 (100), 239 (25), 226 (6), 198 (4), 182 (10); HREIMS Calcd. for [C_17_H_20_N_2_O_2_]: 284.1525. Found: 284.1521.

*10-Valeryl-11-methoxy-3-methyl-5,6-dihydro-β-carboline* (**5**). Off white crystals (MeOH); mp 268–269 °C; IR (KBr) ν_max_: 3,421 (indole N-H), 1,662 (ketone C=O) cm^-1^; ^1^H-NMR (CDCl_3_): δ 7.87 (1H, s, H-9), 6.89 (1H, s, H-12), 3.87 (3H, s, OCH_3_), 3.76 (2H, t, *J* = 8.4 Hz, H-5), 2.95 (2H, t, *J* = 7.4 Hz, H-2'), 2.85 (2H, t, *J* = 8.4 Hz, H-6), 2.43 (3H, s, H-14), 1.68 (2H, quintet, *J* = 7.4 Hz, H-3'), 1.36 (2H, sextet, *J* = 7.4 Hz, H-4'), 0.91 (3H, t, *J* = 7.4 Hz, H-5'); ^13^C-NMR (CDCl_3_): δ 203.1 (C-1'), 158.1 (C-3), 157.7 (C-11), 140.2 (C-13), 129.6 (C-2), 125.2 (C-8), 123.4 (C-9), 119.5 (C-10), 118.6 (C-7), 93.8 (C-12), 55.7 (OCH_3_), 47.6 (C-5), 44.0 (C-2'), 26.4 (C-3'), 22.6 (C-4'), 21.8 (C-14), 19.6 (C-6), 14.1 (C-5'); EIMS *m/z* (rel. int.%): 298 [M^+^] (42), 256 (7), 241 (100), 239 (61), 226 (4), 198 (3), 182 (19), 57 (34); HREIMS Calcd. for [C_18_H_22_N_2_O_2_]: 298.1681. Found: 298.1694.

*10-Hexanoyl-11-methoxy-3-methyl-5,6-dihydro-β-carboline* (**6**). Off white crystals (MeOH); mp 271–272 °C; IR (KBr) ν_max_: 3,420 (indole N-H), 1,665 (ketone C=O) cm^-1^; ^1^H-NMR (CDCl_3_, 400 MHz): δ 7.85 (1H, s, H-9), 6.88 (1H, s, H-12), 3.87 (3H, s, OCH_3_), 3.76 (2H, t, *J* = 8.3 Hz, H-5), 2.95 (2H, t, *J* = 7.4 Hz, H-2'), 2.82 (2H, t, *J* = 8.3 Hz, H-6), 2.36 (3H, s, H-14), 1.67 (2H, quintet, *J* = 7.4 Hz, H-3'), 1.31 (2H, m, H-4'), 1.23 (2H, m, H-5'), 0.88 (3H, t, *J* = 7.4 Hz, H-6'); ^13^C-NMR (CDCl_3_, 100 MHz): δ 203.2 (C-1'), 158.0* (C-3), 157.8* (C-11), 141.2 (C-13), 129.8 (C-2), 125.4 (C-8), 123.2 (C-9), 120.2 (C-10), 118.7 (C-7), 94.2 (C-12), 55.6 (OCH_3_), 47.5 (C-5), 44.1 (C-2'), 31.5 (C-4'), 25.1 (C-3'), 22.7 (C-5'), 21.8 (C-14), 19.6 (C-6), 14.0 (C-6'); EIMS *m/z* (rel. int.%): 312 [M^+^] (30), 256 (14), 241 (100), 239 (47), 226 (8), 198 (8), 182 (22); HREIMS Calcd. for [C_19_H_24_N_2_O_2_]: 312.1838. Found: 312.1831.

*10-Heptanoyl-11-methoxy-3-methyl-5,6-dihydro-β-carboline* (**7**). Off white crystals (MeOH); mp 274–275 °C; IR (KBr) ν_max_: 3,418 (indole N-H), 1,664 (ketone C=O) cm^-1^; ^1^H-NMR (CDCl_3_): δ 7.89 (1H, s, H-9), 6.88 (1H, s, H-12), 3.89 (3H, s, OCH_3_), 3.75 (2H, t, *J* = 8.3 Hz, H-5), 2.95 (2H, t, *J* = 7.4 Hz, H-2'), 2.86 (2H, t, *J* = 8.3 Hz, H-6), 2.45 (3H, s, H-14), 1.66 (2H, quintet, *J* = 7.4 Hz, H-3'), 1.28 (4H, m, H-4' and H-5'), 1.24 (2H, m, H-6'), 0.86 (3H, t, *J* = 7.4 Hz, H-7'); ^13^C-NMR (CDCl_3_): δ 203.2 (C-1'), 158.8 (C-3), 158.1 (C-11), 141.3 (C-13), 129.9 (C-2), 125.5 (C-8), 123.5 (C-9), 120.1 (C-10), 118.6 (C-7), 94.1 (C-12), 55.8 (OCH_3_), 47.5 (C-5), 43.7 (C-2'), 31.7 (C-5'), 29.7 (C-4'), 25.0 (C-3'), 22.5 (C-6'), 21.7 (C-14), 19.5 (C-6), 14.0 (C-7'); EIMS *m/z* (rel. int.%): 326 [M^+^] (68), 256 (16), 241 (100), 239 (20), 226 (4), 198 (4), 182 (5); HREIMS Calcd. for [C_20_H_26_N_2_O_2_]: 326.1994. Found: 326.1991.

*10-Capryloyl-11-methoxy-3-methyl-5,6-dihydro-β-carboline* (**8**). Off white crystals (MeOH); mp 275–276 °C; IR (KBr) ν_max_: 3,413 (indole N-H), 1,667 (ketone C=O) cm^-1^; ^1^H-NMR (C_5_D_5_N): δ 12.41 (1H, s, indole NH), 8.24 (1H, s, H-9), 7.27 (1H, s, H-12), 3.87 (2H, t, *J* = 8.8 Hz, H-5), 3.76 (3H, s, OCH_3_), 3.10 (2H, t, *J* = 7.2 Hz, H-2'), 2.92 (3H, s, H-14), 2.91 (2H, t, *J* = 8.8 Hz, H-6), 1.83 (2H, quintet, *J* = 7.2 Hz, H-3'), 1.36 (2H, quintet, *J* = 7.2 Hz, H-4'), 1.26 (6H, m, H-5' to H-7'), 0.81 (3H, t, *J* = 7.2 Hz, H-8'); ^13^C-NMR (C_5_D_5_N): δ 202.5 (C-1'), 157.0 (C-3 and C-11), 141.0 (C-13), 131.9 (C-2), 125.6 (C-8), 123.2 (C-9), 118.3* (C-10), 118.1* (C-7), 94.6 (C-12), 55.7 (OCH_3_), 47.5 (C-5), 43.9 (C-2'), 31.9 (C-6'), 29.6** (C-5'), 29.5** (C-4'), 25.1 (C-3'), 22.8 (C-7'), 21.9 (C-14), 19.4 (C-6), 14.2 (C-8'); EIMS *m/z* (rel. int.%): 340 [M^+^] (40), 256 (13), 241 (100), 149 (21), 57 (36); HREIMS Calcd. for [C_21_H_28_N_2_O_2_]: 340.2150. Found: 340.2170. 

*10-Nonanoyl-11-methoxy-3-methyl-5,6-dihydro-β-carboline* (**9**). Off white crystals (MeOH); mp 276–277 °C; IR (KBr) ν_max_: 3,420 (indole N-H), 1,662 (ketone C=O) cm^-1^; ^1^H-NMR (CDCl_3_): δ 7.92 (1H, s, H-9), 6.88 (1H, s, H-12), 3.90 (3H, s, OCH_3_), 3.80 (2H, t, *J* = 8.2 Hz, H-5), 2.96 (2H, t, *J* = 7.5 Hz, H-2'), 2.79 (2H, t, *J* = 8.2 Hz, H-6), 2.39 (3H, s, H-14), 1.69 (2H, quintet, *J* = 7.5 Hz, H-3'), 1.34 (2H, quintet, *J* = 7.5 Hz, H-4'), 1.23 (8H, m, H-5' to H-8'), 0.85 (3H, t, *J* = 7.5 Hz, H-9'); ^13^C-NMR (CDCl_3_) δ: 202.5 (C-1'), 158.7 (C-3), 158.2 (C-11), 141.1 (C-13), 130.2 (C-2), 125.5 (C-8), 123.4 (C-9), 120.1 (C-10), 118.6 (C-7), 94.4 (C-12), 55.6 (OCH_3_), 47.3 (C-5), 44.1 (C-2'), 31.9 (C-7'), 29.8* (C-6'), 29.7* (C-5'), 29.5 (C-4'), 25.2 (C-3'), 22.9 (C-8'), 21.7 (C-14), 19.5 (C-6), 14.1 (C-9'); EIMS *m/z* (rel. int.%): 354 [M^+^] (46), 341 (6), 256 (17), 255 (13), 242 (17), 241 (100), 239 (54), 226 (4), 182 (12); HREIMS Calcd. for [C_22_H_30_N_2_O_2_]: 354.2307. Found: 354.2313. 

*10-Decanoyl-11-methoxy-3-methyl-5,6-dihydro-β-carboline* (**10**). Off white crystals (MeOH); mp 279–280 °C; IR (KBr) ν_max_: 3,420 (indole N-H), 1,662 (ketone C=O) cm^-1^; ^1^H-NMR (CDCl_3_): δ 7.88 (1H, s, H-9), 6.86 (1H, s, H-12), 3.87 (3H, s, OCH_3_), 3.72 (2H, t, *J* = 8.2 Hz, H-5), 2.94 (2H, t, *J* = 7.2 Hz, H-2'), 2.83 (2H, t, *J* = 8.2 Hz, H-6), 2.40 (3H, s, H-14), 1.66 (2H, quintet, *J* = 7.2 Hz, H-3'), 1.34 (2H, quintet, *J* = 7.2 Hz, H-4'), 1.24 (10H, m, H-5' to H-9'), 0.85 (3H, t, *J* = 7.2 Hz, H-10'); ^13^C-NMR (CDCl_3_): δ 202.5 (C-1'), 159.0 (C-3), 158.0 (C-11), 141.5 (C-13), 130.5 (C-2), 125.5 (C-8), 123.5 (C-9), 120.2 (C-10), 118.7 (C-7), 94.4 (C-12), 55.6 (OCH_3_), 47.5 (C-5), 44.0 (C-2'), 32.0 (C-8'), 29.8 (C-5' to C-7'), 29.5 (C-4'), 25.3 (C-3'), 22.9 (C-9'), 21.8 (C-14), 19.7 (C-6), 14.2 (C-10'); EIMS *m/z* (rel. int.%): 368 [M^+^] (56), 256 (17), 241 (100), 239 (54), 226 (7), 198 (6), 182(12); HREIMS Calcd. for [C_23_H_32_N_2_O_2_]: 368.2464. Found: 368.2477.

*12-Acetyl-11-methoxy-3-methyl-5,6-dihydro-β-carboline* (**11**). Off white crystals (MeOH); mp 255–256 °C; IR (KBr) ν_max_: 3,414 (indole N-H), 1,637 (ketone C=O) cm^-1^; ^1^H-NMR (CDCl_3_): δ 10.60 (1H, br.s, indole NH), 7.70 (1H, d, *J* = 8.8 Hz, H-9), 6.85 (1H, d, *J* = 8.8 Hz, H-10), 4.00 (3H, s, OCH_3_), 3.82 (2H, t, *J* = 8.5 Hz, H-5), 2.80 (2H, t, *J* = 8.5 Hz, H-6), 2.71(3H, s , H-2'), 2.36 (3H, s, H-14); ^13^C-NMR (CDCl_3_,): δ 200.5 (C-1'), 159.9 (C-11), 157.6 (C-3), 137.4 (C-13), 129.6 (C-2), 127.0 (C-9), 121.1 (C-8), 116.1 (C-7), 110.4 (C-12), 105.7 (C-10), 56.3 (OCH_3_), 48.0 (C-5), 33.6 (C-2'), 21.8 (C-14), 19.1 (C-6); EIMS *m/z* (rel. int.%): 256 [M^+^] (100), 241 (39), 226 (4), 198 (7), 182 (5); HREIMS Calcd. for [C_15_H_16_N_2_O_2_]: 256.1212. Found: 256.1216.

*12-Propionyl-11-methoxy-3-methyl-5,6-dihydro-β-carboline* (**12**). Off white crystals (MeOH); mp 257–258 °C; IR (KBr) ν_max_: 3,414 (indole N-H), 1,637 (ketone C=O) cm^-1^; ^1^H-NMR (CDCl_3_): δ 10.67 (1H, br.s, indole NH), 7.70 (1H, d, *J* = 8.7 Hz, H-9), 6.86 (1H, d, *J* = 8.7 Hz, H-10), 4.00 (3H, s, OCH_3_), 3.82 (2H, t, *J* = 8.3 Hz, H-5), 3.14 (2H, q, *J* = 7.2 Hz, H-2'), 2.80 (2H, t, *J* = 8.3 Hz, H-6), 2.35 (3H, s, H-14), 1.20 (3H, t, *J* = 7.2 Hz, H-3'); ^13^C-NMR (CDCl_3_): δ 203.7 (C-1'), 159.7 (C-11), 157.5 (C-3), 137.6 (C-13), 129.6 (C-2), 126.7 (C-9), 121.2 (C-8), 116.0 (C-7), 110.1 (C-12), 105.8 (C-10), 56.3 (OCH_3_), 48.1 (C-5), 38.3 (C-2'), 21.9 (C-14), 19.2 (C-6), 8.4 (C-3'); EIMS *m/z* (rel. int.%): 270 [M^+^] (100), 255 (44), 241 (21), 226 (4), 213 (5), 198 (9) 182 (9); HREIMS Calcd. for [C_16_H_18_N_2_O_2_]: 270.1368. Found: 270.1357.

*12-Butyryl-11-methoxy-3-methyl-5,6-dihydro-β-carboline* (**13**). Off white crystals (MeOH); mp 260–261 °C; IR (KBr) ν_max_: 3,418 (indole N-H), 1,636 (ketone C=O) cm^-1^; ^1^H-NMR (CDCl_3_): δ 10.66 (1H, br.s, indole NH), 7.70 (1H, d, *J* = 8.7 Hz, H-9), 6.86 (1H, d, *J* = 8.7 Hz, H-10), 4.00 (3H, s, OCH_3_), 3.82 (2H, t, *J* =8.4 Hz, H-5), 3.08 (2H, t, *J* = 7.3 Hz, H-2'), 2.80 (2H, t, *J* = 8.4 Hz, H-6), 2.36 (3H, s, H-14), 1.75 (2H, sextet, *J* = 7.3 Hz, H-3'), 1.00 (3H, t, *J* = 7.3 Hz, H-4'); ^13^C-NMR (CDCl_3_): δ 203.4 (C-1'), 159.7 (C-11), 157.7 (C-3), 137.4 (C-13), 129.6 (C-2), 126.8 (C-9), 121.2 (C-8), 116.1 (C-7), 110.2 (C-12), 105.8 (C-10), 56.4 (OCH_3_), 47.8 (C-5), 45.9 (C-2'), 21.9 (C-14), 19.2 (C-6), 17.9 (C-3'), 14.0 (C-4'); EIMS *m/z* (rel. int.%): 284 [M^+^] (100), 269 (12), 255 (63), 241 (25), 226 (5), 213 (6), 198 (12), 182 (17); HREIMS Calcd. for [C_17_H_20_N_2_O_2_]: 284.1525. Found: 284.1533.

*12-Valeryl-11-methoxy-3-methyl-5,6-dihydro-β-carboline* (**14**). Off white crystals (MeOH); mp 263–264 °C; IR (KBr) ν_max_: 3,416 (indole N-H), 1,638 (ketone C=O) cm^-1^; ^1^H-NMR (CDCl_3_): δ 10.65 (1H, br.s, indole NH), 7.70 (1H, d, *J* = 8.8 Hz, H-9), 6.86 (1H, d, *J* = 8.8 Hz, H-10), 4.00 (3H, s, OCH_3_), 3.82 (2H, t, *J* = 8.1 Hz, H-5), 3.11 (2H, t, *J* = 7.3 Hz, H-2'), 2.80 (2H, t, *J* = 8.1 Hz, H-6), 2.36 (3H, s, H-14), 1.70 (2H, quintet, *J* = 7.3 Hz, H-3'), 1.42 (2H, sextet, *J* = 7.3, H-4'), 0.96 (3H, t, *J* = 7.3 Hz, H-5'); ^13^C-NMR (CDCl_3_): δ 203.4 (C-1'), 159.6 (C-11), 157.7 (C-3), 137.6 (C-13), 129.5 (C-2), 126.8 (C-9), 121.2 (C-8), 116.0 (C-7), 110.2 (C-12), 105.8 (C-10), 56.3 (OCH_3_), 47.9 (C-5), 44.8 (C-2'), 26.7 (C-3'), 22.6 (C-4'), 21.8 (C-14), 19.1 (C-6), 14.1 (C-5'); EIMS *m/z* (rel. int.%): 298 [M^+^] (100), 283 (9), 255 (52), 241 (28), 226 (4), 213 (6), 198 (8), 182 (12); HREIMS Calcd. for [C_18_H_22_N_2_O_2_]: 298.1681. Found: 298.1692.

*12-Hexanoyl-11-methoxy-3-methyl-5,6-dihydro-β-carboline* (**15**). Off white crystals (MeOH); mp 265–266 °C; IR (KBr) ν_max_: 3,412 (indole N-H), 1,637 (ketone C=O) cm^-1^; ^1^H-NMR (CDCl_3_): δ 10.66 (1H, br.s, indole NH), 7.70 (1H, d, *J* = 8.6 Hz, H-9), 6.87 (1H, d, *J* = 8.6 Hz, H-10), 4.00 (3H, s, OCH_3_), 3.83 (2H, t, *J* = 8.0 Hz, H-5), 3.10 (2H, t, *J* = 7.1 Hz, H-2'), 2.81 (2H, t, *J* = 8.0 Hz, H-6), 2.37 (3H, s, H-14), 1.70 (2H, quintet, *J* = 7.1 Hz, H-3'), 1.37 (2H, m, H-4'), 1.29 (2H, m, H-5'), 0.91 (3H, t, *J* = 7.1 Hz, H-6'); ^13^C-NMR (CDCl_3_): δ 203.4 (C-1'), 159.7 (C-11), 157.9 (C-3), 137.7 (C-13), 129.5 (C-2), 126.8 (C-9), 121.1 (C-8), 116.4 (C-7), 110.2 (C-12), 105.9 (C-10), 56.3 (OCH_3_), 47.6 (C-5), 45.1 (C-2'), 31.7 (C-4'), 24.2 (C-3'), 22.6 (C-5'), 21.5 (C-14), 19.1 (C-6), 14.0 (C-6'); EIMS *m/z* (rel. int.%): 312 [M^+^] (100), 297 (12), 255 (62) , 241 (39), 226 (7), 213 (8), 198 (9), 182 (9); HREIMS Calcd. for [C_19_H_24_N_2_O_2_]: 312.1838. Found: 312.1826.

*12-Heptanoyl-11-methoxy-3-methyl-5,6-dihydro-β-carboline* (**16**). Off white crystals (MeOH); mp 268–269 °C; IR (KBr) ν_max_: 3,417 (indole N-H), 1,637 (ketone C=O) cm^-1^; ^1^H-NMR (CDCl_3_): δ 10.73 (1H, br.s, indole NH), 7.72 (1H, d, *J* = 8.8 Hz, H-9), 6.88 (1H, d, *J* = 8.8 Hz, H-10), 4.01 (3H, s, OCH_3_), 3.85 (2H, t, *J* = 8.4 Hz, H-5), 3.10 (2H, t, *J* = 7.4 Hz, H-2'), 2.85 (2H, t, *J* = 8.4 Hz, H-6), 2.42 (3H, s, H-14), 1.71 (2H, quintet, *J* = 7.4 Hz, H-3'), 1.39 (2H, quintet, *J* = 7.4, H-4'), 1.27 (4H, m, H-5' and H-6'), 0.88 (3H, t, *J* = 7.4 Hz, H-7'); ^13^C-NMR (CDCl_3_): δ 203.2 (C-1'), 159.5 (C-11), 157.8 (C-3), 137.7 (C-13), 129.6 (C-2), 127.0 (C-9), 121.3 (C-8), 116.2 (C-7), 110.2 (C-12), 106.1 (C-10), 56.4 (OCH_3_), 46.8 (C-5), 45.2 (C-2'), 31.5 (C-5'), 28.9 (C-4'), 25.0 (C-3'), 22.5 (C-6'), 21.6 (C-14), 19.1 (C-6), 14.0 (C-7'); EIMS *m/z* (rel. int.%): 326 [M^+^] (100), 269 (6), 255 (43), 241 (20), 226 (4), 213 (6), 198 (5), 182 (8), 57 (29); HREIMS Calcd. for [C_20_H_26_N_2_O_2_]: 326.1994. Found: 326.1999.

*12-Capryloyl-11-methoxy-3-methyl-5,6-dihydro-β-carboline* (**17**). Off white crystals (MeOH); mp 271–272 °C; IR (KBr) ν_max_: 3,413 (indole N-H), 1,637 (ketone C=O) cm^-1^; ^1^H-NMR (CDCl_3_): δ 10.70 (1H, br.s, indole NH), 7.72 (1H, d, *J* = 8.8 Hz, H-9), 6.88 (1H, d, *J* = 8.8 Hz, H-10), 4.00 (3H, s, OCH_3_), 3.85 (2H, t, *J* = 8.1 Hz, H-5), 3.10 (2H, t, *J* = 7.2 Hz, H-2'), 2.84 (2H, t, *J* = 8.1 Hz, H-6), 2.42 (3H, s, H-14), 1.70 (2H, quintet, *J* = 7.2 Hz, H-3'), 1.34 (2H, quintet, *J* = 7.2, H-4'), 1.26 (6H, m, H-5' to H-7'), 0.85 (3H, t, *J* = 7.2 Hz, H-8'); ^13^C-NMR (CDCl_3_): δ 203.4 (C-1'), 159.6 (C-11), 157.7 (C-3), 137.9 (C-13), 129.3 (C-2), 126.6 (C-9), 121.3 (C-8), 116.6 (C-7), 110.1 (C-12), 106.1 (C-10), 56.1 (OCH_3_), 47.1 (C-5), 45.1 (C-2'), 31.9 (C-6'), 29.5* (C-5'), 29.4* (C-4'), 24.7 (C-3'), 22.7 (C-7'), 21.4 (C-14), 19.4 (C-6), 14.0 (C-8'); EIMS *m/z* (rel. int.%): 340[M^+^] (100), 255 (43), 241 (20), 239 (28), 198 (8), 182 (15); HREIMS Calcd. for [C_21_H_28_N_2_O_2_]: 340.2150. Found: 340.2170.

*12-Nonanoyl-11-methoxy-3-methyl-5,6-dihydro-β-carboline* (**18**). Off white crystals (MeOH); mp 273–274 °C; IR (KBr) ν_max_: 3,414 (indole N-H), 1,638 (ketone C=O) cm^-1^; ^1^H-NMR (CDCl_3_): δ 10.77 (1H, br.s, indole NH), 7.72 (1H, d, *J* = 8.8 Hz, H-9), 6.88 (1H, d, *J* = 8.8 Hz, H-10), 4.00 (3H, s, OCH_3_), 3.87 (2H, t, *J* = 8.1 Hz, H-5), 3.10 (2H, t, *J* = 7.3 Hz, H-2'), 2.88 (2H, t, *J* = 8.1 Hz, H-6), 2.46 (3H, s, H-14), 1.71 (2H, quintet, *J* = 7.3 Hz, H-3'), 1.40 (2H, quintet, *J* = 7.3, H-4'), 1.26 (8H, m, H-5' to C-8'), 0.86 (3H, t, *J* = 7.3 Hz, H-9'); ^13^C-NMR (CDCl_3_): δ 203.5 (C-1'), 159.7 (C-11), 157.9 (C-3), 137.8 (C-13), 129.4 (C-2), 126.7 (C-9), 121.3 (C-8), 116.5 (C-7), 110.1 (C-12), 106.1 (C-10), 56.1 (OCH_3_), 47.3 (C-5), 45.1 (C-2'), 31.9 (C-7'), 29.7 (C-6'), 29.5* (C-5'), 29.4* (C-4'), 24.6 (C-3'), 22.9 (C-8'), 21.6 (C-14), 19.3 (C-6), 14.1 (C-9'); EIMS *m/z* (rel. int.%): 354 [M^+^] (100), 339 (5), 269 (5), 255 (31), 241 (18), 226 (4), 213 (5), 198 (3), 182 (5); HREIMS Calcd. for [C_22_H_30_N_2_O_2_]: 354.2307. Found: 354.2313. 

*12-Decanoyl-11-methoxy-3-methyl-5,6-dihydro-β-carboline* (**19**). Off white crystals (MeOH); mp 275–276 °C; IR (KBr) ν_max_: 3,415 (indole N-H), 1,638 (ketone C=O) cm^-1^; ^1^H-NMR (CDCl_3_): δ 10.70 (1H, br.s, indole NH), 7.70 (1H, d, *J* = 8.8 Hz, H-9), 6.87 (1H, d, *J* = 8.8 Hz, H-10), 4.00 (3H, s, OCH_3_), 3.84 (2H, t, *J* = 8.2 Hz, H-5), 3.10 (2H, t, *J* = 7.3 Hz, H-2'), 2.83 (2H, t, *J* = 8.2 Hz, H-6), 2.40 (3H, s, H-14), 1.71 (2H, quintet, *J* = 7.3 Hz, H-3'), 1.34 (2H, m, H-4'), 1.25 (10H, m, H-5' to H-9'), 0.86 (3H, t, *J* = 7.3 Hz, H-10'); ^13^C-NMR (CDCl_3_): δ 203.4 (C-1'), 159.8 (C-11), 158.3 (C-3), 137.9 (C-13), 129.4 (C-2), 126.9 (C-9), 121.1 (C-8), 116.8 (C-7), 110.2 (C-12), 106.0 (C-10), 56.3 (OCH_3_), 47.1 (C-5), 45.2 (C-2'), 31.9 (C-8'), 29.7* (C-7'), 29.6* (C-6'), 29.5* (C-5'), 29.3 (C-4'), 24.5 (C-3'), 22.7 (C-9'), 21.2 (C-14), 19.1 (C-6), 14.1 (C-10'); EIMS *m/z* (rel. int.%): 368 [M^+^] (100), 353 (12) 269 (4) 255 (27), 241 (22), 226 (4), 213 (8), 198 (9), 182 (22), 57 (28); HREIMS Calcd. for [C_23_H_32_N_2_O_2_]: 368.2464. Found: 368.2452. 

*2-Acetyl-3-(2-acetamidoethyl)-7-methoxyindole* (**20**). Colorless crystalline solid (CHCl_3_-MeOH, 1:1); mp 152–153 °C; IR (KBr) ν_max_: 3,436, 3,314, 3,270 (indolic and amide N-H), 1,690, 1,640 (ketone and amide C=O) cm^-1^; ^1^H-NMR (CDCl_3_): δ 8.86 (1H, br.s, indole NH) 7.54 (1H, d, *J* = 8.8 Hz, H-5), 6.79 (1H, dd, *J* = 8.8 and 1.8 Hz, H-6), 6.75 (1H, br.s, H-8), 5.85 (1H, br.t, CONH), 3.83 (3H, s, OCH_3_), 3.54 (2H, q, *J* = 6.8 Hz, H-2'), 3.28 (2H, t, *J* = 6.8 Hz, H-1'), 2.60 (3H, s, 2-COCH_3_), 1.90 (3H, s, H-2''); ^13^C-NMR (CDCl_3_): δ 189.8 (2-COCH_3_), 170.3 (C-1''), 160.0 (C-7), 137.3 (C-9), 131.8 (C-2), 122.8 (C-4), 122.0 (C-5), 120.9 (C-3), 112.4 (C-6), 93.4 (C-8), 55.5 (OCH_3_), 40.8 (C-2'), 28.1 (2-COCH_3_), 25.3 (C-1'), 23.3 (C-2''); EIMS *m/z* (rel. int.%): 274 [M^+^] (14), 215 (100), 203 (42), 202 (57), 188 (46), 160 (37), 145 (22); HREIMS Calcd. for [C_15_H_18_N_2_O_3_]: 274.1317. Found: 274.1322.

*2-Acetyl-3-(2-propionylamidoethyl)-7-methoxyindole* (**21**). Colorless crystalline solid (CHCl_3_-MeOH, 1:1); mp 156–157 °C; IR (KBr) ν_max_: 3,432, 3,315, 3,274 (indolic and amide N-H), 1,691, 1,637 (ketone and amide C=O) cm^-1^; ^1^H-NMR (CDCl_3_): δ 8.80 (1H, br.s, indole NH), 7.55 (1H, d, *J* = 8.8 Hz, H-5), 6.79 (1H, dd, *J* = 8.8 and 2.0 Hz, H-6), 6.75 (1H, d, *J* = 2.0 Hz, H-8), 5.73 (1H, br.t, CONH), 3.84 (3H, s, OCH_3_), 3.55 (2H, q, *J* = 6.9 Hz, H-2'), 3.28 (2H, t, *J* = 6.9 Hz, H-1'), 2.61 (3H, s, 2-COCH_3_), 2.12 (2H, q, *J* = 7.6 Hz, H-2''), 1.08 (3H, t, *J* = 7.6 Hz, H-3''); ^13^C-NMR (CDCl_3_): δ 189.7 (2-COCH_3_), 174.0 (C-1''), 160.0 (C-7), 137.2 (C-9), 131.8 (C-2), 122.8 (C-4), 122.1 (C-5), 120.9 (C-3), 112.4 (C-6), 93.4 (C-8), 55.5 (OCH_3_), 40.7 (C-2'), 29.7 (C-2''), 28.1 (2-COCH_3_), 25.4 (C-1'), 9.7 (C-3''); EIMS *m/z* (rel. int.%): 288 [M^+^] (13), 215 (100), 203 (32), 202 (51) 188 (19), 57 (53); HREIMS Calcd. for [C_16_H_20_N_2_O_3_]: 288.1474. Found: 288.1477.

*2-Acetyl-3-(2-butyrylamidoethyl)-7-methoxyindole* (**22**). Colorless crystalline solid (CHCl_3_-MeOH, 1:1); mp 161–162 °C; IR (KBr) ν_max_: 3,430, 3,315, 3,258 (indolic and amide N-H), 1,695, 1,631(ketone and amide C=O) cm^-1^; ^1^H-NMR (CDCl_3_): δ 8.80 (1H, br.s, indole NH), 7.56 (1H, d, *J* = 8.8 Hz, H-5), 6.79 (1H, dd, *J* = 8.8 and 2.1 Hz, H-6), 6.75 (1H, d, *J* = 2.1 Hz, H-8), 5.74 (1H, br.t, CONH), 3.84 (3H, s, OCH_3_), 3.55 (2H, q, *J* = 6.7 Hz, H-2'), 3.28 (2H, t, *J* = 6.7 Hz, H-1'), 2.62 (3H, s, 2-COCH_3_), 2.07 (2H, t, *J* = 7.4 Hz, H-2''), 1.59 (2H, sextet, *J* = 7.4 Hz, H-3''^´^), 0.89 (3H, t, *J* = 7.4 Hz, H-4''); ^13^C-NMR (CDCl_3_): δ 189.8 (2-COCH_3_), 173.2 (C-1''), 160.0 (C-7), 137.3 (C-9), 131.9 (C-2), 122.8 (C-4), 122.1 (C-5), 120.9 (C-3), 112.4 (C-6), 93.4 (C-8), 55.5 (OCH_3_), 40.6 (C-2'), 38.7 (C-2''), 28.1 (2-COCH_3_), 25.4 (C-1'), 19.0 (C-3''), 13.8 (C-4''); EIMS *m/z* (rel. int.%): 302 [M^+^] (6), 259 (3) 215 (100), 203 (33), 202 (39), 188 (20), 160 (20); HREIMS Calcd. for [C_17_H_22_N_2_O_3_]: 302.1630. Found: 302.1642.

*2-Acetyl-3-(2-valerylamidoethyl)-7-methoxyindole* (**23**). Colorless crystalline solid (CHCl_3_-MeOH, 1:1); mp 163–164 °C; IR (KBr) ν_max_: 3,420, 3,314, 3,260 (indolic and amide N-H), 1,692, 1,633 (ketone and amide C=O) cm^-1^; ^1^H-NMR (CDCl_3_): δ 8.81 (1H, br.s, indole NH), 7.55 (1H, d, *J* = 8.8 Hz, H-5), 6.79 (1H, dd, *J* = 8.8 and 2.0 Hz, H-6), 6.75 (1H, d, *J* = 2.0 Hz, H-8), 5.74 (1H, br.t, CONH), 3.84 (3H, s, OCH_3_), 3.54 (2H, q, *J* = 6.9 Hz, H-2'), 3.28 (2H, t, *J* = 6.9 Hz, H-1'), 2.62 (3H, s, 2-COCH_3_), 2.09 (2H, t, *J* = 7.3 Hz, H-2''), 1.54 (2H, quintet, *J* = 7.3 Hz, H-3''^´^), 1.26 (2H, sextet, *J* = 7.3 Hz, H-4''), 0.87 (3H, t, *J* = 7.3 Hz, H-5''); ^13^C-NMR (CDCl_3_): δ 189.7 (2-COCH_3_), 173.3 (C-1''), 160.0 (C-7), 137.2 (C-9), 131.9 (C-2), 122.9 (C-4), 122.1 (C-5), 120.9 (C-3), 112.4 (C-6), 93.4 (C-8), 55.5 (OCH_3_), 40.6 (C-2'), 36.5 (C-2''), 28.1 (2-COCH_3_), 27.7 (C-3''), 25.5 (C-1'), 22.4 (C-4''), 13.8 (C-5''); EIMS *m/z* (rel. int.%): 316 [M^+^] (9), 273 (2), 215 (100), 203 (32), 202 (38), 188 (17), 160 (18), 85 (33), 57 (66); HREIMS Calcd. for [C_18_H_24_N_2_O_3_]: 316.1787. Found: 316.1802. 

*2-Acetyl-3-(2-hexanoylamidoethyl)-7-methoxyindole* (**24**). Colorless crystalline solid (CHCl_3_-MeOH, 1:1); mp 167–168 °C; IR (KBr) ν_max_: 3,428, 3,317, 3,270 (indolic and amide N-H), 1,692, 1,633 (ketone and amide C=O) cm^-1^; ^1^H-NMR (CDCl_3_): δ 8.80 (1H, br.s, indole NH) 7.55 (1H, d, *J* = 8.8 Hz, H-5), 6.79 (1H, dd, *J* = 8.8 and 2.0 Hz, H-6), 6.75 (1H, d, *J* = 2.0 Hz, H-8), 5.73 (1H, br.t, CONH), 3.84 (3H, s, OCH_3_), 3.55 (2H, q, *J* = 6.9 Hz, H-2'), 3.28 (2H, t, *J* = 6.9 Hz, H-1'), 2.61 (3H, s, 2-COCH_3_), 2.08 (2H, t, *J* = 6.7 Hz, H-2''), 1.56 (2H, quintet, *J* = 6.7 Hz, H-3''^´^), 1.25 (4H, m, H-4'' and H-5''), 0.86 (3H, t, *J* = 6.7 Hz, H-6''); ^13^C-NMR (CDCl_3_): δ 189.7 (2-COCH_3_), 173.3 (C-1''), 160.0 (C-7), 137.3 (C-9), 131.9 (C-2), 122.9 (C-4), 122.1 (C-5), 120.9 (C-3), 112.4 (C-6), 93.5 (C-8), 55.5 (OCH_3_), 40.7 (C-2'), 36.8 (C-2''), 31.5 (C-4''), 28.1 (2-COCH_3_), 25.5 (C-1'), 25.3 (C-3''), 22.4 (C-5''), 13.9 (C-6''); EIMS *m/z* (rel. int.%): 330 [M^+^] (8), 287 (2), 215 (100), 203 (30), 202 (22), 188 (12), 160 (7), 145 (5), 99 (6); HREIMS Calcd. for [C_19_H_26_N_2_O_3_]: 330.1943. Found: 330.1956.

*2-Acetyl-3-(2-heptanoylamidoethyl)-7-methoxyindole* (**25**). Colorless crystalline solid (CHCl_3_-MeOH, 1:1); mp 171–172 °C; IR (KBr) ν_max_: 3,430, 3,314, 3,274 (indolic and amide N-H), 1,691, 1,637 (ketone and amide C=O) cm^-1^; ^1^H-NMR (CDCl_3_): δ 8.79 (1H, br.s, indole NH), 7.56 (1H, d, *J* = 8.8 Hz, H-5), 6.79 (1H, dd, *J* = 8.8 and 2.1 Hz, H-6), 6.75 (1H, d, *J* = 2.1 Hz, H-8), 5.72 (1H, br.t, CONH), 3.84 (3H, s, OCH_3_), 3.54 (2H, q, *J* = 7.0 Hz, H-2'), 3.28 (2H, t, *J* = 7.0 Hz, H-1'), 2.61 (3H, s, 2-COCH_3_), 2.08 (2H, t, *J* = 7.0 Hz, H-2''), 1.55 (2H, quintet, *J* = 7.0 Hz, H-3''^´^), 1.25 (6H, m, H-4'' to H-6''), 0.85 (3H, t, *J* = 7.0 Hz, H-7''); ^13^C-NMR (CDCl_3_): δ 189.7 (2-COCH_3_), 173.3 (C-1''), 160.0 (C-7), 137.3 (C-9), 131.9 (C-2), 122.9 (C-4), 122.1 (C-5), 120.9 (C-3), 112.4 (C-6), 93.5 (C-8), 55.5 (OCH_3_), 40.7 (C-2'), 36.8 (C-2''), 31.5 (C-5''), 28.9 (C-4''), 28.1 (2-COCH_3_), 25.6* (C-3''), 25.5* (C-1'), 22.4 (C-6''), 14.0 (C-7''); EIMS *m/z* (rel. int.%): 344 [M^+^] (8), 301 (3), 215 (100), 203 (27), 202 (18), 188 (15), 160 (12), 145 (7), 113 (4); HREIMS Calcd. for [C_20_H_28_N_2_O_3_]: 344.2100. Found: 344.2110.

*2-Acetyl-3-(2-capryloylamidoethyl)-7-methoxyindole* (**26**). Colorless crystalline solid (CHCl_3_-MeOH, 1:1); mp 172–173 °C; IR (KBr) ν_max_: 3,436, 3,315, 3,270 (indolic and amide N-H), 1,695, 1,637 (ketone and amide C=O) cm^-1^; ^1^H-NMR (CDCl_3_): δ 8.85 (1H, br.s, indole NH), 7.55 (1H, d, *J* = 8.8 Hz, H-5), 6.79 (1H, dd, *J* = 8.8 and 2.2 Hz, H-6), 6.74 (1H, d, *J* = 2.2 Hz, H-8), 5.77 (1H, br.t, CONH), 3.83 (3H, s, OCH_3_), 3.54 (2H, q, *J* = 6.9 Hz, H-2'), 3.28 (2H, t, *J* = 6.9 Hz, H-1'), 2.61 (3H, s, 2-COCH_3_), 2.08 (2H, t, *J* = 7.3 Hz, H-2''), 1.54 (2H, quintet, *J* = 7.3 Hz, H-3''^´^), 1.24 (8H, m, H-4'' to H-7''), 0.85 (3H, t, *J* = 7.3 Hz, H-8''); ^13^C-NMR (CDCl_3_): δ 189.7 (2-COCH_3_), 173.3 (C-1''), 160.0 (C-7), 137.3 (C-9), 131.9 (C-2), 122.9 (C-4), 122.1 (C-5), 120.9 (C-3), 112.4 (C-6), 93.5 (C-8), 55.5 (OCH_3_), 40.7 (C-2'), 36.8 (C-2''), 31.7 (C-6''), 29.3 (C-5''), 29.0 (C-4''), 28.1 (2-COCH_3_), 25.6* (C-3''), 25.5* (C-1'), 22.6 (C-7''), 14.0 (C-8''); EIMS *m/z* (rel. int.%): 358 [M^+^] (7), 315 (2), 215 (100), 203 (27), 202 (21), 160 (10), 145 (4), 127 (4), 57 (24); HREIMS Calcd. for [C_21_H_30_N_2_O_3_]: 358.2256. Found: 358.2261.

*2-Acetyl-3-(2-nonanoylamidoethyl)-7-methoxyindole* (**27**). Colorless crystalline solid (CHCl_3_-MeOH, 1:1); mp 178–179 °C ; IR (KBr) ν_max_: 3,440, 3,320, 3,275 (indolic and amide N-H), 1,690, 1,634 (ketone and amide C=O) cm^-1^; ^1^H-NMR (CDCl_3_): δ 8.80 (1H, br.s, indole NH), 7.55 (1H, d, *J* = 8.8 Hz, H-5), 6.79 (1H, dd, *J* = 8.8 and 1.9 Hz, H-6), 6.75 (1H, d, *J* = 1.9 Hz, H-8), 5.75 (1H, br.t, CONH), 3.84 (3H, s, OCH_3_), 3.54 (2H, q, *J* = 6.9 Hz, H-2'), 3.28 (2H, t, *J* = 6.9 Hz, H-1'), 2.62 (3H, s, 2-COCH_3_), 2.08 (2H, t, *J* = 7.1 Hz, H-2''), 1.55 (2H, quintet, *J* = 7.1 Hz, H-3''^´^), 1.24 (10H, m, H-4'' to H-8''), 0.85 (3H, t, *J* = 7.1 Hz, H-9''); ^13^C-NMR (CDCl_3_): δ 189.7 (2-COCH_3_), 173.4 (C-1''), 160.0 (C-7), 137.2 (C-9), 131.9 (C-2), 122.8 (C-4), 122.1 (C-5), 120.9 (C-3), 112.4 (C-6), 93.4 (C-8), 55.5 (OCH_3_), 40.6 (C-2'), 36.8 (C-2''), 31.8 (C-7''), 29.3 (C-5''and C-6''), 29.1 (C-4''), 28.1 (2-COCH_3_), 25.6* (C-3''), 25.5* (C-1'), 22.6 (C-8''), 14.1 (C-9''); EIMS *m/z* (rel. int.%): 372 [M^+^] (3), 215 (100), 203 (29), 202 (35), 188 (17), 174 (19), 160 (18), 145 (12), 57 (35), 55 (28); HREIMS Calcd. for [C_22_H_32_N_2_O_3_]: 372.2413. Found 372.2426.

*2-Acetyl-3-(2-decanoylamidoethyl)-7-methoxyindole* (**28**). Colorless crystalline solid (CHCl_3_-MeOH, 1:1); mp 183–184 °C; IR (KBr) ν_max_: 3,423, 3,317, 3,274 (indolic and amide N-H), 1,690, 1,637 (ketone and amide C=O) cm^-1^; ^1^H-NMR (CDCl_3_): δ 8.90 (1H, br.s, indole NH), 7.55 (1H, d, *J* = 8.9 Hz, H-5), 6.78 (1H, dd, *J* = 8.9 and 1.7 Hz, H-6), 6.74 (1H, d, *J* = 1.7 Hz, H-8), 5.79 (1H, br.t, CONH), 3.83 (3H, s, OCH_3_), 3.54 (2H, q, *J* = 6.9 Hz, H-2'), 3.28 (2H, t, *J* = 6.9 Hz, H-1'), 2.61 (3H, s, 2-COCH_3_), 2.08 (2H, t, *J* = 6.9 Hz, H-2''), 1.54 (2H, quintet, *J* = 6.9 Hz, H-3''^´^), 1.25 (12H, m, H-4'' to H-9''), 0.85 (3H, t, *J* = 6.9 Hz, H-10''); ^13^C-NMR (CDCl_3_): δ 189.7 (2-COCH_3_), 173.4 (C-1''), 160.0 (C-7), 137.3 (C-9), 131.9 (C-2), 122.8 (C-4), 122.1 (C-5), 120.9 (C-3), 112.4 (C-6), 93.4 (C-8), 55.6 (OCH_3_), 40.7 (C-2'), 36.8 (C-2''), 31.8 (C-8''), 29.4* (C-7''), 29.3 (C-5'' and C-6''), 29.2* (C-4''), 28.1 (2-COCH_3_), 25.6** (C-3''), 25.5** (C-1'), 22.7 (C-9''), 14.1 (C-10''); EIMS m/z (rel. int.%): 386 [M^+^] (10), 243 (3), 215 (100), 203 (30), 202 (14), 188 (12), 174 (9), 145 (4), 160 (7); HREIMS Calcd. for [C_23_H_34_N_2_O_3_]: 386.2569. Found: 386.2580.

*2-Acetyl-3-(2-capryloylamidoethyl)-8-capryloyl-7-methoxyindole* (**29**). Colorless crystalline solid (CHCl_3_-MeOH, 1:1); mp 196–198 °C; IR (KBr) ν_max_: 3,436, 3,290 (indolic and amide N-H), 1,692, 1,637 (ketone and amide C=O) cm^-1^; ^1^H-NMR (CDCl_3_): δ 11.02 (1H, br.s, indole NH), 7.87 (1H, d, *J* = 9.0 Hz, H-5), 6.89 (1H, d, *J* = 9.0 Hz, H-6), 6.17 (1H, br.t, CONH), 4.02 (3H, s, OCH_3_), 3.52 (2H, q, *J* = 6.4 Hz, H-2'), 3.28 (2H, t, *J* = 6.4 Hz, H-1'), 3.08 (2H, t, *J* = 7.2 Hz, H-2'''), 2.62 (3H, s, 2-COCH_3_), 2.05 (2H, t, *J* = 6.5 Hz, H-2''), 1.71 (2H, quintet, *J* = 7.2 Hz, H-3'''^´^), 1.56 (2H, quintet, *J* = 6.5 Hz, H-3''^´^), 1.34 (2H, m, H-4'''), 1.28 (6H, m, H-5''' to H-7'''), 1.24 (8H, m, H-4'' to H-7''), 0.86 (3H, t, *J* = 7.2 Hz, H-8'''), 0.84 (3H, t, *J* = 6.5 Hz, H-8''); ^13^C-NMR (CDCl_3_): δ 203.0 (C-1'''), 190.7 (2-COCH_3_), 173.5 (C-1''), 161.0 (C-7), 137.2 (C-9), 132.4 (C-2), 128.1 (C-5), 123.7 (C-4), 121.4 (C-3), 109.7 (C-8), 106.8 (C-6), 56.4 (OCH_3_), 45.1 (C-2'''), 41.0 (C-2'), 36.8 (C-2''), 31.8 (C-6'''), 31.6 (C-6''), 29.5 (C-5'''), 29.3* (C-5''), 29.2* (C-4'''), 29.0 (C-4''), 28.1 (2-COCH_3_), 25.6 (C-1'), 24.5 (C-3''') 24.3 (C-3''), 22.6 (C-7'''), 22.5 (C-7''), 14.1 (C-8'''), 14.0 (C-8''); EIMS *m/z* (rel. int.%): 484 [M^+^] (5), 441 (2), 341 (100), 329 (10), 328 (9), 270 (8), 257 (15), 242 (20), 230 (15), (63); HREIMS Calcd. for [C_29_H_44_N_2_O_4_ ]: 484.3301. Found: 484.3303.

*2-Acetyl-3-(2-nonanoylamidoethyl)-8-nonanoyl-7-methoxyindole* (**30**). Colorless crystalline solid (CHCl_3_-MeOH, 1:1); mp 199–200 °C; IR (KBr) ν_max_: 3,432, 3,295 (indolic and amide N-H), 1,690, 1,637 (ketone and amide C=O) cm^-1^; ^1^H-NMR (CDCl_3_): δ 11.03 (1H, br.s, indole NH), 7.86 (1H, d, *J* = 8.8 Hz, H-5), 6.90 (1H, d, *J* = 8.8 Hz, H-6), 6.19 (1H, br.t, CONH), 4.02 (3H, s, OCH_3_), 3.51 (2H, q, *J* = 6.4 Hz, H-2'), 3.28 (2H, t, *J* = 6.4 Hz, H-1'), 3.08 (2H, t, *J* = 6.9 Hz, H-2'''), 2.62 (3H, s, 2-COCH_3_), 2.05 (2H, t, *J* = 6.7 Hz, H-2''), 1.70 (2H, quintet, *J* = 6.9 Hz, H-3'''^´^), 1.53 (2H, quintet, *J* = 6.7 Hz, H-3''^´^), 1.34 (2H, m, H-4'''), 1.28 (8H, m, H-5''' to H-8'''), 1.26 (10H, m, H-4'' to H-8''), 0.86 (3H, t, *J* = 6.9 Hz, H-9'''), 0.85 (3H, t, *J* = 6.7 Hz, H-9''); ^13^C-NMR (CDCl_3_): δ 203.1 (C-2'''), 190.7 (2-COCH_3_), 173.5 (C-1''), 161.0 (C-7), 137.2 (C-9), 132.4 (C-2), 128.1 (C-5), 123.7 (C-4), 121.4 (C-3), 109.7 (C-8), 106.8 (C-6), 56.4 (OCH_3_), 45.1 (C-2'''), 40.9 (C-2'), 36.8 (C-2''), 31.8 (C-7'' and C-7'''), 29.6 (C-4'''), 29.5 (C-5'''and C-6'''), 29.3 (C-4'' to C-6''), 28.1 (2-COCH_3_), 25.6 (C-1'), 24.5 (C-3'''), 24.3 (C-3''), 22.7 (C-8'' and C-8'''), 14.1 (C-9'' and C-9'''); EIMS *m/z* (rel. int.%): 512[M^+^] (15), 469 (20), 355 (100), 343 (8), 342 (6), 270 (8), 257 (12), 242 (14), 230 (12); HREIMS Calcd. for [C_31_H_48_N_2_O_4_]: 512.3602. Found: 512.3591.

*2-Acetyl-3-(2-decanoylamidoethyl)-8-decanoyl-7-methoxyindole* (**31**). Colorless crystalline solid (CHCl_3_-MeOH, 1:1); mp 201–202 °C; IR (KBr) ν_max_: 3,436, 3,295 (indolic and amide N-H), 1,691, 1,637 (ketone and amide C=O) cm^-1^; ^1^H-NMR (CDCl_3_): δ 11.03 (1H, br.s, indole NH), 7.86 (1H, d, *J* = 8.8 Hz, H-5), 6.89 (1H, d, *J* = 8.8 Hz, H-6), 6.18 (1H, br.t, CONH), 4.02 (3H, s, OCH_3_), 3.52 (2H, q, *J* = 6.4 Hz, H-2'), 3.28 (2H, t, *J* = 6.4 Hz, H-1'), 3.08 (2H, t, *J* = 7.0 Hz, H-2'''), 2.62 (3H, s, 2-COCH_3_), 2.05 (2H, t, *J* = 6.7 Hz, H-2''), 1.70 (2H, quintet, *J* = 7.0 Hz, H-3'''^´^), 1.51 (2H, quintet, *J* = 6.7 Hz, H-3''^´^), 1.34 (2H, m, H-4'''), 1.26 (22H, m, H-4'' to H-9'' and H-5''' to H-9'''), 0.86 (3H, t, *J* = 7.0 Hz, H-10'''), 0.85 (3H, t, *J* = 6.7 Hz, H-10''); ^13^C-NMR (CDCl_3_): δ 203.0 (C-2'''), 190.7 (2-COCH_3_), 173.4 (C-1''), 161.0 (C-7), 137.2 (C-9), 132.4 (C-2), 128.1 (C-5), 123.7 (C-4), 121.4 (C-3), 109.7 (C-8), 106.8 (C-6), 56.4 (OCH_3_), 45.1 (C-2'''), 40.9 (C-2'), 36.8 (C-2''), 31.9 (C-8'''), 31.8 (C-8''), 29.6* (C-4'''), 29.5** (C-5'''and C-6'''), 29.4* (C-7''), 29.3** (C-5'' and C-6''), 29.2* (C-4'' and C-7'''), 28.1 (2-COCH_3_), 25.6 (C-1'), 24.5 (C-3'''), 24.3 (C-3''), 22.7 (C-9'' and C-9'''), 14.1 (C-10'' and C-10'''); (*,** values may be interchanged for a given compound); EIMS *m/z* (rel. int.%): 540 [M^+^] (15), 497 (20), 370 (85), 369 (100), 357 (47), 356 (24), 270 (25), 257 (50), 242 (60), 230 (35); HREIMS Calcd. for [C_33_H_52_N_2_O_4_]: 540.3967. Found: 540.3977.

## Conclusions

In summary, a mild and efficient one pot synthesis of a series of benzene ring acylated analogues of harmaline was carried out at room temperature. The problem of the sensitive imine-enamine functionality in harmaline was tackled by increasing the quantity of AlCl_3_. The 10-substituted regioselectivity of the reaction was observed with increase with the carbon chain length in the acylating agents. The present procedure offers several advantages including high yield, shorter reaction time, ambient conditions, operational simplicity and minimum environmental effects.
